# Blocking autophagy improves the anti-tumor activity of afatinib in lung adenocarcinoma with activating *EGFR* mutations *in vitro* and *in vivo*

**DOI:** 10.1038/s41598-017-04258-8

**Published:** 2017-07-04

**Authors:** Xiangxiang Hu, Si Shi, Huan Wang, Xiaochen Yu, Qian Wang, Shanshan Jiang, Dianwen Ju, Li Ye, Meiqing Feng

**Affiliations:** Department of Microbiological & Biochemical Pharmacy, School of Pharmacy, Fudan University, Shanghai, 201203 China

## Abstract

Afatinib, a second-generation tyrosine kinase inhibitor (TKI), has been approved for the treatment of advanced *EGFR*-mutant non-small cell lung cancer (NSCLC). However, afatinib’s clinical application is still hampered by acquired resistance. Recently, autophagy is considered as an important mechanism of resistance to TKI. Herein, we investigated the autophagy induction as well as its influence on anti-lung adenocarcinoma activity of afatinib in two activating *EGFR*-mutants H1975 and H1650 cells. First, Growth inhibition and caspase-dependent apoptosis were observed in afatinib-treated H1975 and H1650 cells. Then we confirmed afatinib-induced autophagy in H1975 and H1650 cells. Importantly, autophagy inhibition using chloroquine (CQ) and 3-MA enhanced the cytotoxicity of afatinib, elucidating the cytoprotective role of autophagy in lung adenocarcinoma therapy with afatinib. Further study suggested that Akt/mTOR and Erk signaling pathways were involved in afatinib-induced autophagy, and reactive oxygen species (ROS) acted as an intracellular transducer regulating both autophagy and apoptosis in afatinib-treated H1975 and H1650 cells. Moreover, the *in vivo* experiment in xenograft model using H1975 cell line confirmed the enhanced anti-lung adenocarcinoma efficacy of afatinib when combined with autophagy inhibitor CQ. Thus, blocking autophagy may be a promising strategy to overcome resistance and increase sensitivity to afatinib in lung adenocarcinoma harboring activating *EGFR* mutations.

## Introduction

Lung cancer, the leading course of tumor-related mortality, is responsible for nearly 1.4 million deaths each year worldwide^1, 2^. Non-small cell lung cancer (NSCLC) makes up the majority (about 80%) of all lung cancer^3, 4^, and the primary type of NSCLC is lung adenocarcinoma which accounts for 40% of all lung cancer patients. Epidermal growth factor receptor (EGFR) is overexpressed in 80% of NSCLCs, and its mutations are considered as important drivers of NSCLC. EGFR activation triggers the phosphorylation of the inner tyrosine kinase portion of the receptor, then leads to cell proliferation and apoptosis suppression via activating intracellular pathway^5^. Despite the development in the diagnosis and treatment, the rate of overall 5-year survival of lung adenocarcinoma after diagnosis still remains very low^6, 7^. Therefore, there is a significant need to find novel therapeutic approaches for treating lung adenocarcinoma.

Afatinib, a second-generation tyrosine kinase inhibitor(TKI), is an oral and irreversible ErbB family blocker^8^. Compared with first-generation EGFR TKIs such as erlotinib and gefitinib which are reversible TKIs, afatinib can irreversibly block signaling from ErbB family dimers through covalently binding to EGFR (ErbB1), human epidermal growth factor receptor 2 (HER2/ErbB2), ErbB3 and ErbB4^9, 10^. In consideration of its pan-ErbB inhibition and activity against both sensitizing and resistance *EGFR* mutations, afatinib has been approved for the treatment of advanced *EGFR*-mutant NSCLCs including common (exon 19 deletion) or acquired (T790M) reversible EGFR-TKIs resistance mutations in several countries^11–13^.

Despite improved clinical efficacy in the treatment for advanced NSCLC with activating *EGFR* mutations, afatinib’s clinical application is still hampered by acquired resistance and adverse events^11^. It is suggested that combination therapy may be useful in terms of overcoming resistance and improving tolerability to afatinib^14^. Autophagy is an intracellular catabolic process that maintains cellular energetic balance through the degradation of proteins and organelles in lysosomes^15, 16^, and can be upregulated by environmental stimuli including chemotherapeutic agents, oxidative stress and nutrient starvation^17–20^. Although autophagy has a role as a double-edged sword in disease and health^21^, many studies show that it serves a cytoprotective function particularly in cancer treatment^22–24^. Recently, autophagy is considered as an important mechanism of resistance to TKI including afatinib^25^. It has been proved that autophagy is involved in the induction of erlotinib resistance in tongue squamous cell carcinoma^26^, and blocking autophagic flux in pancreatic cancer cell lines sensitizes EGFR-TKI-induced non-apoptotic cell death^27^. However, the role of autophagy in advanced *EGFR*-mutant NSCLC therapy using afatinib has not been clearly elucidated.

In this study, two activating *EGFR*-mutants H1975 (L858R/T790M) and H1650 (exon 19 deletion) cells were selected to study the activities of cytotoxicity and autophagy induction of afatinib. We found that afatinib not only induced growth inhibition and caspase-dependent apoptosis, but also activated autophagic response in H1975 and H1650 cells, and autophagy played a survival role in this process. The mechanism by which afatinib initiated autophagy was also explored. In addition, in tumor xenograft model using H1975 cell line performed in nude mice, the combination treatment of afatinib and CQ significantly reduced the tumor volume and tumor weight. Taken together, autophagy plays a cytoprotective role in afatinib therapy and the inhibition of autophagy shows an increased sensitivity of lung adenocarcinoma cells harboring activating *EGFR* mutations to afatinib.

## Results

### Afatinib induces growth inhibition and apoptosis in H1975 and H1650 cells

**Figure 1:**
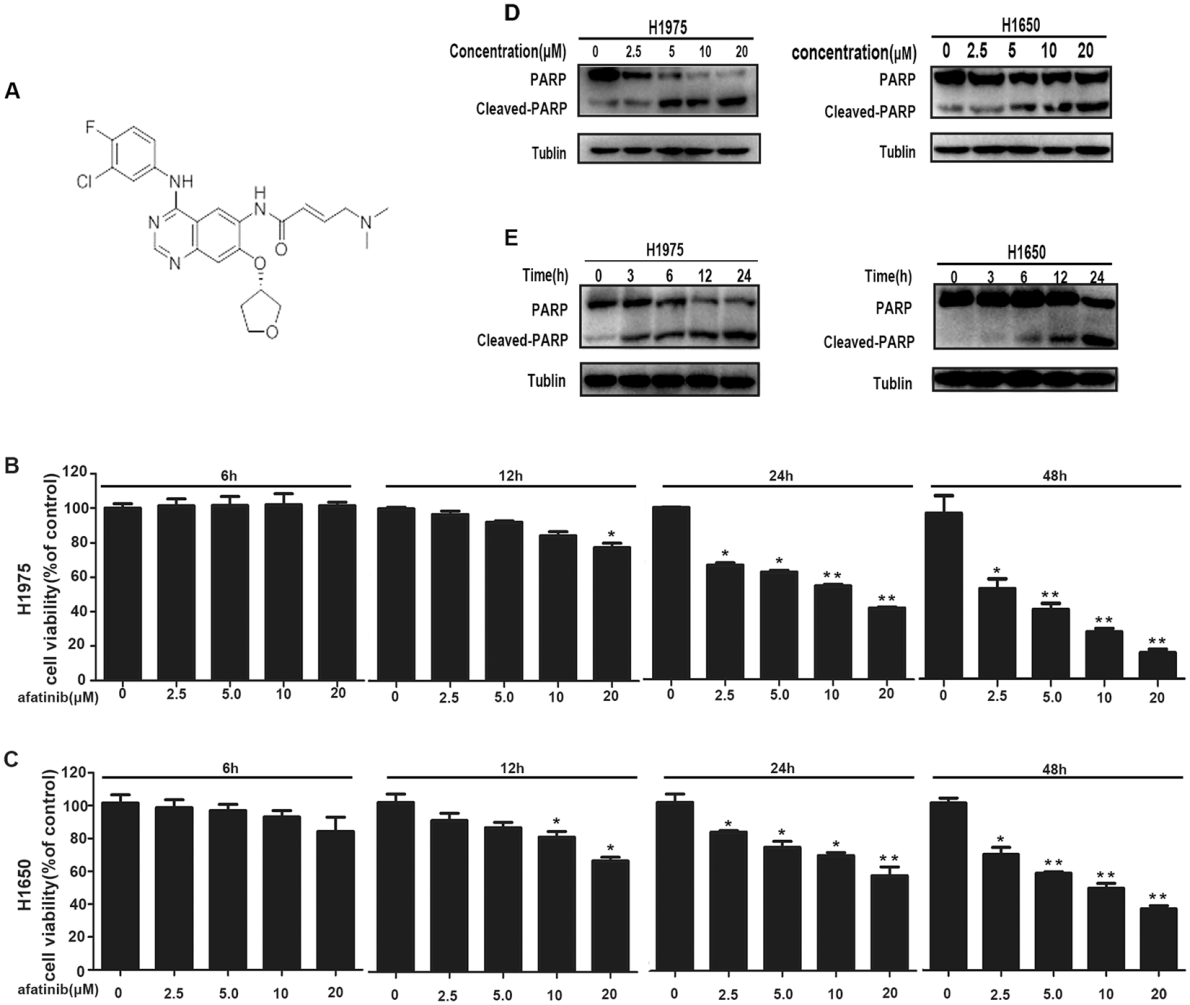
Afatinib induces cytotoxicity and apoptosis in H1975 and H1650 cells. (**A**) The structure formula of afatinib. (**B** and **C**) H1975 and H1650 cells were dealt with various concentrations of afatinib for indicated time, then MTT assay was used to measure cell viability. All these data were represented as mean ± SD (*P < 0.05, **P < 0.01, ***P < 0.001 versus control). (**D**) H1975 and H1650 cells were incubated with various concentrations of afatinib for 24 h, then the levels of PARP and cleaved-PARP were measured by western blot analysis. These blots were cropped with Photoshop CS6. (**E**) H1975 and H1650 cells were treated with 10 μM afatinib for different time, then western blot analysis was performed to assess the levels of PARP and cleaved-PARP. These blots were cropped with Photoshop CS6.

Afatinib is a anilino-quinazoline derivative that can covalently bind to Cys 773 of EGFR, Cys 805 of HER2 and Cys803 of ErbB4, and its structure is shown in Fig. 1A. H1650 and H1975 cells were dealt with afatinib (0–20 μM) for indicated time, then the MTT assay was used to detect the cell viability. We can see from the Fig. 1B and C that H1975 and H1650 cells viability could be suppressed by afatinib dose- and time-dependently. Meanwhile, the results of western blot illustrated that the expression of cleaved-PARP increased in afatinib-treated cells (Fig. 1D and E), indicating that the apoptosis was induced by afatinib in H1650 and H1975 cells. These data show that afatinib can induce growth inhibition and apoptosis in lung adenocarcinoma cells with activating *EGFR* mutations through a dose- and time-dependent way.

### Afatinib-triggered apoptosis is partially caspase 3-dependent in H1975 and H1650 cells

**Figure 2:**
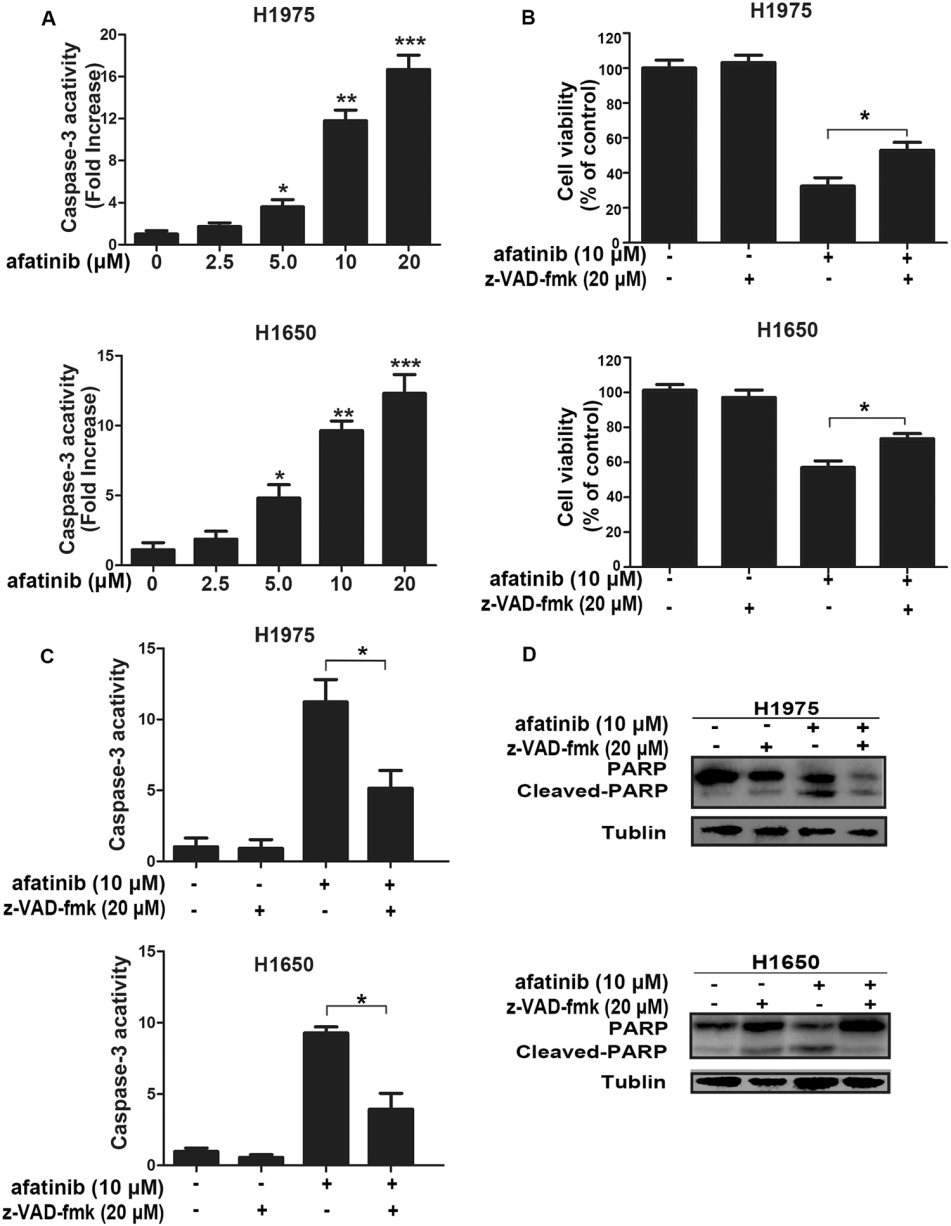
Afatinib-induced apoptosis is partially caspase 3-dependent in H11975 and H1650 cells. (**A**) H1975 and H1650 cells were stimulated by indicated concentrations of afatinib for 48 h, then caspase 3 activity was detected by the caspase 3 activity kit. Data were represented as mean ± SD (*P < 0.05, **P < 0.01, ***P < 0.001 versus control) (**B**–**D**) H1975and H1650 cells were treated with 10 μM afatinib in the absence or presence of 20 μM z-VAD-fmk for 48 h. (**B**) Cell viability was measured by MTT assay at 570 nm. (**C**) The activity of caspase 3 were determined by the caspase 3 activity kit. All data were represented as mean ± SD (*P < 0.05). (**D**) The levels of PARP and cleaved-PARP were measured by western blot analysis. These blots were cropped with Photoshop CS6.

To further determine whether apoptosis induced by afatinib in H1975 and H1650 cells was correlated to the activation of caspase 3, we used casepase 3 activity assay kit to detect the casepase 3 activity of cells which were treated with different concentrations of afatinib for 24 h. From Fig. 2A, we knew that afatinib dramatically increased casepase 3 activity in a dose-dependent manner in both cells. In addition, a pan-caspase inhibitor benzyloxycarbonyl Val-Ala-Asp (O-methyl)-fluoro-methyl ketone (z-VAD-fmk) was employed to test its effect on cell viability and casepase 3 activity. The results showed that 20 μM of z-VAD-fmk could significantly decrease the cytotoxicity of afatinib in both cell lines (Fig. 2B). Moreover, when cells were co-treated with afatinib and z-VAD-fmk for 48 h, both caspase 3 activity and the level of cleaved-PARP were down-regulated significantly as compared to that of afatinib single treatment (Fig. 2C and D).

These results suggest that afatinib-induced apoptosis in lung adenocarcinoma cells partially depends on caspase 3 activation.

### Afatinib induces autophagy in H1975 and H1650 cells

**Figure 3:**
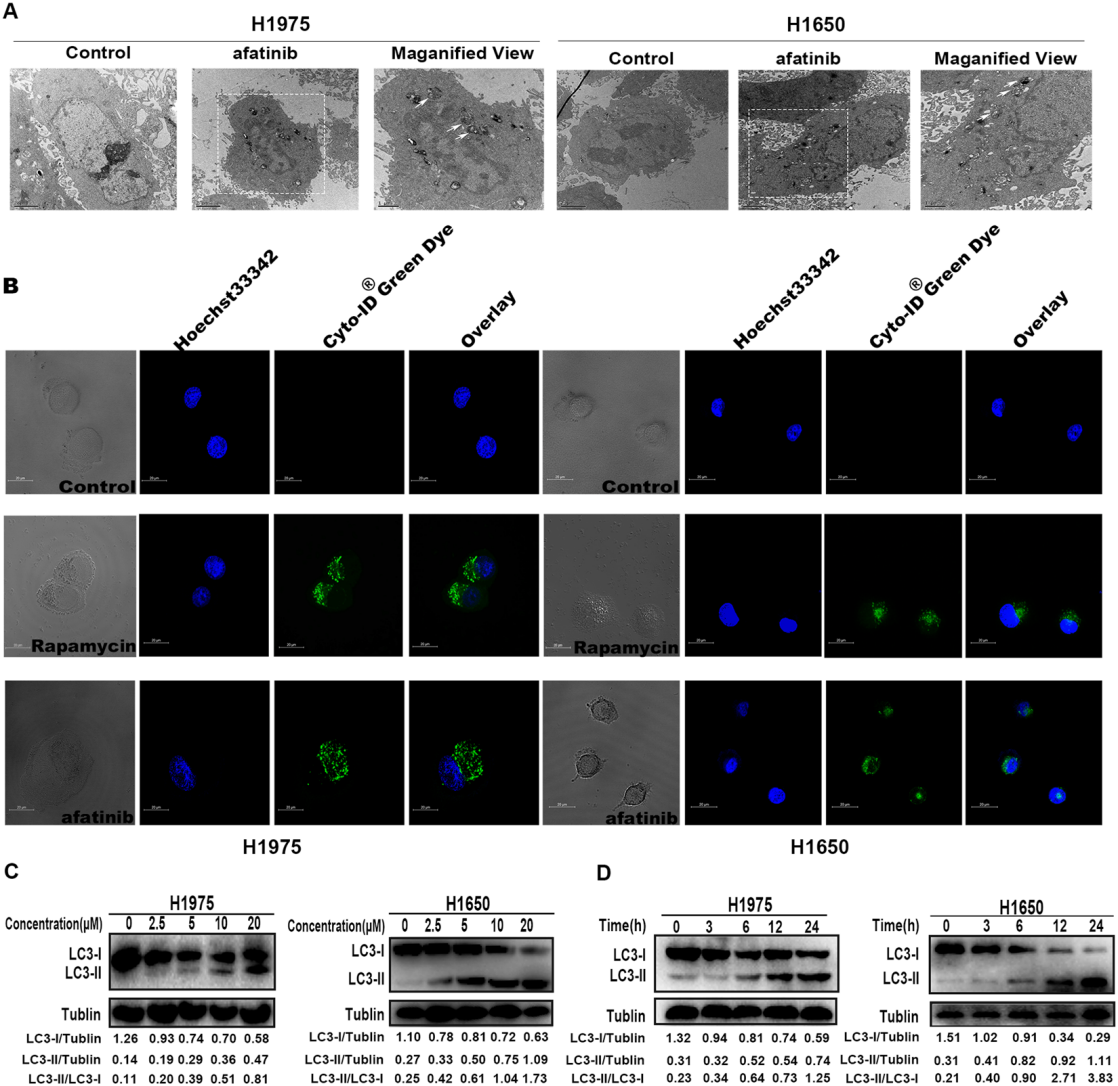
Afatinib induces autophagy in H1975 and H1650 cells. (**A**) H1975 and H1650 cells were treated with 10 μM afatinib for 24 h. The autophagosomes were observed using TEM (white arrows indicated the autophagosomes). (**B**) H1975 and H1650 cells were stimulated by 10 μM afatinib for 24 h, then stained by Cyto-ID^®^ autophagy dye and examined using confocal fluorescent microscopy. 50 nM Rapamycin was used as positive control. (**C**) H1975 and H1650 cells were treated with 2.5, 5.0, 10 and 20 μM of afatinib for 24 h, then the level of LC3-I/II were measured by western blot analysis. These blots were cropped with Photoshop CS6. The concentration of LC3-I and LC3-II were quantified by ImageJ. (**D**) H1975 and H1650 cells were treated with 10 μM afatinib for 0, 3, 6, 12 and 24 h, the production of LC3-I/II were measured with western blot analysis. These blots were cropped with Photoshop CS6. The concentration of LC3-I and LC3-II were quantified by ImageJ.

It has been reported that TKI treatment can induce autophagy in many cancer cells including tongue cancer, pancreatic cancer and chronic myeloid leukaemia (CML), which is now believed to be the main reason for drug resistance^26–28^. We then determined whether autophagy could be induced by afatinib in H1975 and H1650 cells. First, transmission electron microscopy (TEM) analysis was applied to detect the formation of autophagic vacuoles in H1975 and 1650 cells after exposure to afatinib for 24 h. Many double-membrane-enclosed autophagosomes were observed in afatinib-treated cells, while there were little autophagosomes in the negative control (Fig. 3A). Second, Cyto-ID^®^ green dye autophagy detection kit was used to identify the level of LC3-II, which is a protein on the membrane of autophagosomes. After incubated with10 μM afatinib for 24 h, both H1975 and H1650 cells had notable green fluorescence when compared with the untreated cells which displayed no specific fluorescence. Rapamycin (50 nM) treated cells served as the positive control and also had great green fluorescence (Fig. 3B). At last, we determined the conversion of LC3 in the cells treated with different concentrations of afatinib using western blot. The shift of LC3 from the LC3-I to LC3-II is related to the formation of autophagy. From Fig. 3C and D, we found the production of LC3-II increased dose- and time-dependently responding to afatinib treatment.

All of these results strongly support the idea that autophagy can be induced by the afatinib in H1975and H1650 cells.

### Afatinib triggers autophagic flux in H1975 and H1650 cells

**Figure 4:**
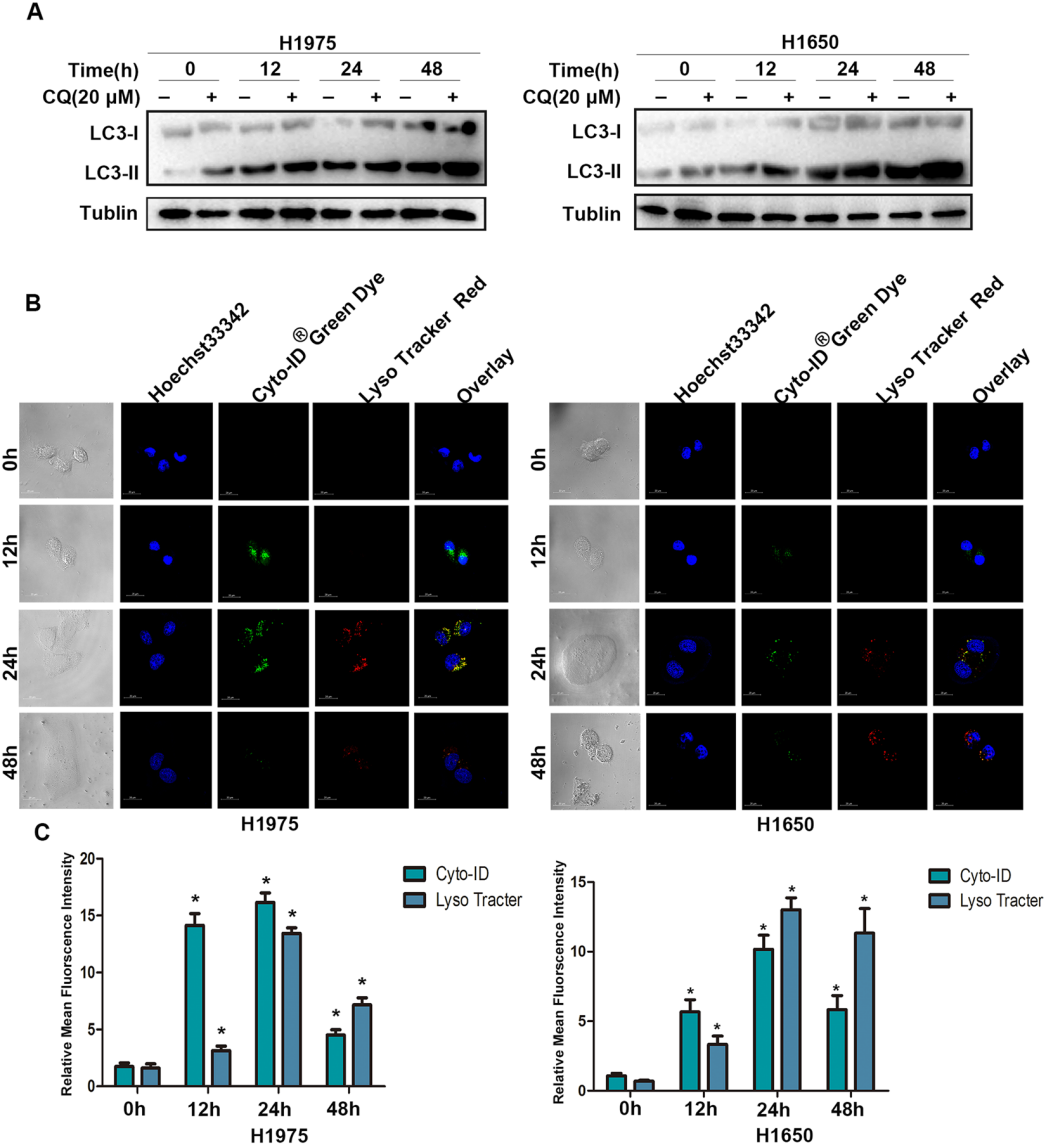
Afatinib triggers autophagic flux in H1975 and H1650 cells. (**A**) H1975 and H1650 cells were simulated by 10 μM afatinib in the presence or absence of 20 μM CQ for 0, 12, 24 and 48 h. The production of LC3-I/II were measured by western blot analysis. These blots were cropped with Photoshop CS6. (**B**) H1975 and H1650 cells were treated with 10 μM of afatinib for 0, 12, 24 and 48 h and the autophagic fluxes were detected with confocal fluorescent microscopy (**C**) The ImageJ was used to perform the quantitation analysis in the Fig. 4B. These data were presented as mean ± SD (*P < 0.05 versus control).

In order to deeply illustrate autophagy triggered by afatinib in H1975 and H1650 cells, we investigated the autophagic flux in the course of afatinib treatment. Chloroquine(CQ), a lysosome inhibitor, can result in the accumulation of autophagosomes and up-regulation of LC3-II by inhibiting the fusion of autophagosomes and lysosomes. Western blot results showed that the aggregation of LC3-II was time-dependent upon afatinib treatment combined with or without CQ. Meanwhile, the LC3-II concentration in the absence of CQ were lower than that in the presence of CQ, notably in 12 h, 24 h, 48 h after afatinib treatment (Fig. 4A), which implied that some LC3 was blocked by CQ to be delivered to lysosomes for degradation. In addition, through confocal fluorescence microscopy (Cyto-ID^®^ and Lyso-Tracker staining), we observed three steps of autophagic flux in afatinib-incubated H1975 and H1650 cells including the production and aggregation of autophagosomes at 12 h (green fluorescence), autophagosome-lysosome fusion at 24 h (yellow fluorescence) and decomposition of autophagosomes by lysosomes at 48 h (red fluorescence) (Fig. 4B and C).

The results strongly support that afatinib induces the autophagic flux in H1975 and H1650 cells.

### Afatinib-induced cytotoxicity and apoptosis can be improved by autophagy inhibition in H1975 and H1650 cells

**Figure 5:**
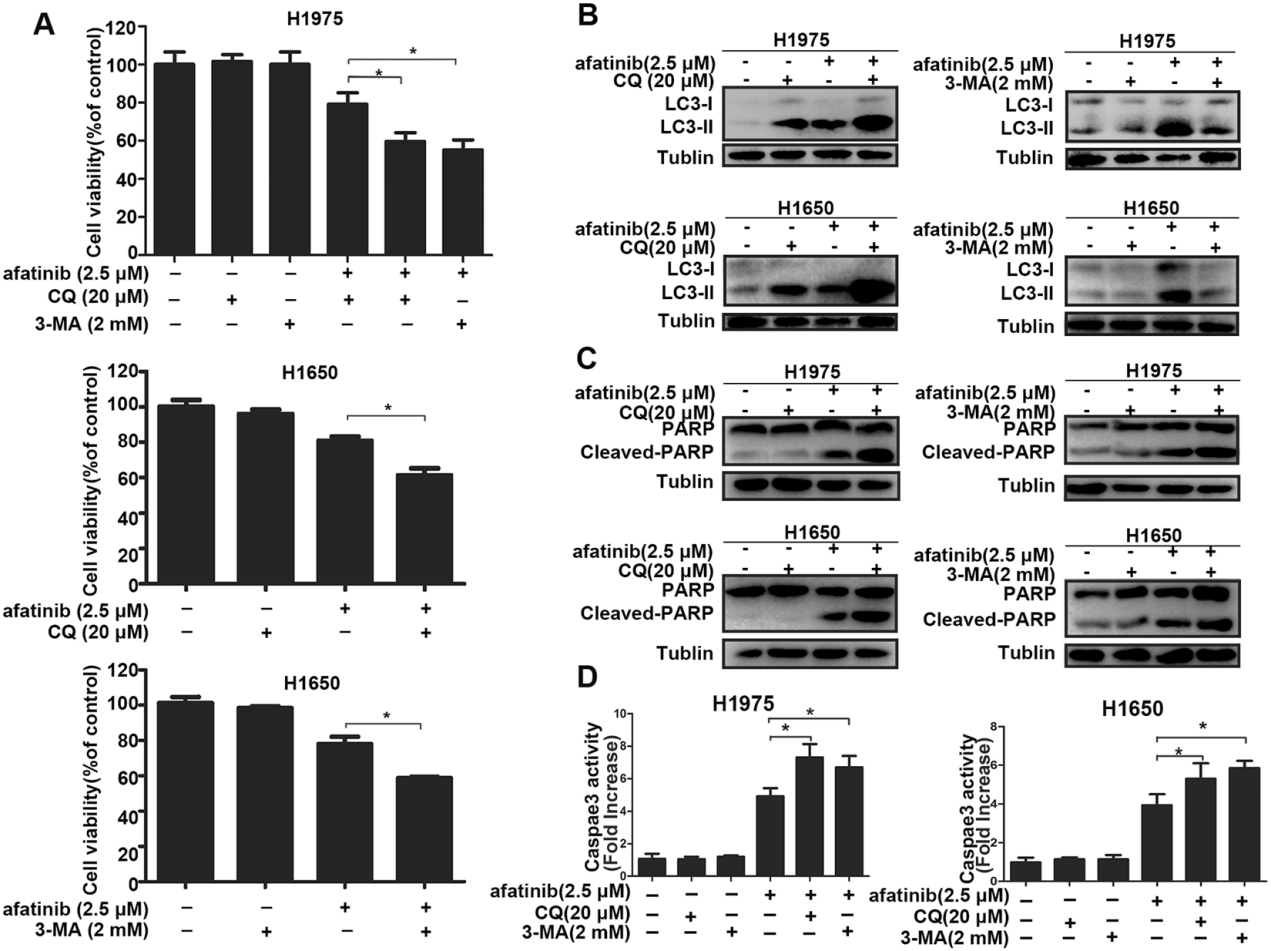
Suppressing of afatinib-induced autophagy promotes the growth inhibition and apoptosis in H1975 and H1650 cells. (**A**–**D**) H1975 and H1650 cells were stimulated by 2.5 μM afatinib in the presence or absence of 20 μM CQ or 2 mM 3-MA for 48 h. (**A**) Cell viability was measured by MTT assay. (**B**) LC3-I/II production were measured by western blot analysis. These blots were cropped with Photoshop CS6. (**C**) Western blot analysis was used to measure the level of PARP and Cleaved-PARP. These blots were cropped with Photoshop CS6. (**D**) The activity of caspase 3 were detected by caspase 3 activity kit. All these data were presented as mean ± SD (*P < 0.05).

Most researches indicate that autophagy serves a cytoprotective role particularly in cancer treatment, and autophagy inhibition can enhance therapy-induced cell death^29, 30^. To determine the role of autophagy caused by afatinib in lung adenocarcinoma cells, two autophagy inhibitor CQ and 3-MA were applied to co-treat cancer cells, and we analyzed their influence on afatinib-induced growth inhibition and apoptosis. It is well known that 3-MA is a PI3K inhibitor that blocks autophagosome aggregation and the transformation of LC3-I to LC3-II, while CQ increases LC3-II level through aggregation of autophagosome. Figure 5A showed that both 3-MA and CQ intensified afatinib-induced cell death in H1975 and H1650 cells. Meanwhile, western blot analysis showed that afatinib-induced autophagy was successfully inhibited by CQ and 3-MA (Fig. 5B). Thus, we got the conclusion that autophagy suppression could increase afatinib-induced cytotoxicity.

We further studied the influence of autophagy on afatinib-induced apoptosis by detecting the changes of the cleaved-PARP level and caspase 3 activity with or without CQ or 3-MA. Western blot analysis revealed that the cleaved-PARP concentrations upon co-treatment with CQ or 3-MA were higher than that upon afatinib alone (Fig. 5C). Moreover, afatinib combined with CQ or 3-MA could increase the activity of caspase 3 significantly in both cells. (Fig. 5D).

All these data illustrate that autophagy has a protective function against the afatinib-triggered cytotoxicity and apoptosis, and autophagy inhibition can improve the anti-cancer effect of afatinib in lung adenocarcinoma cells with activating *EGFR* mutations.

### The afatinib-induced autophagy is related to the Akt/mTOR and Erk signling pathway

**Figure 6:**
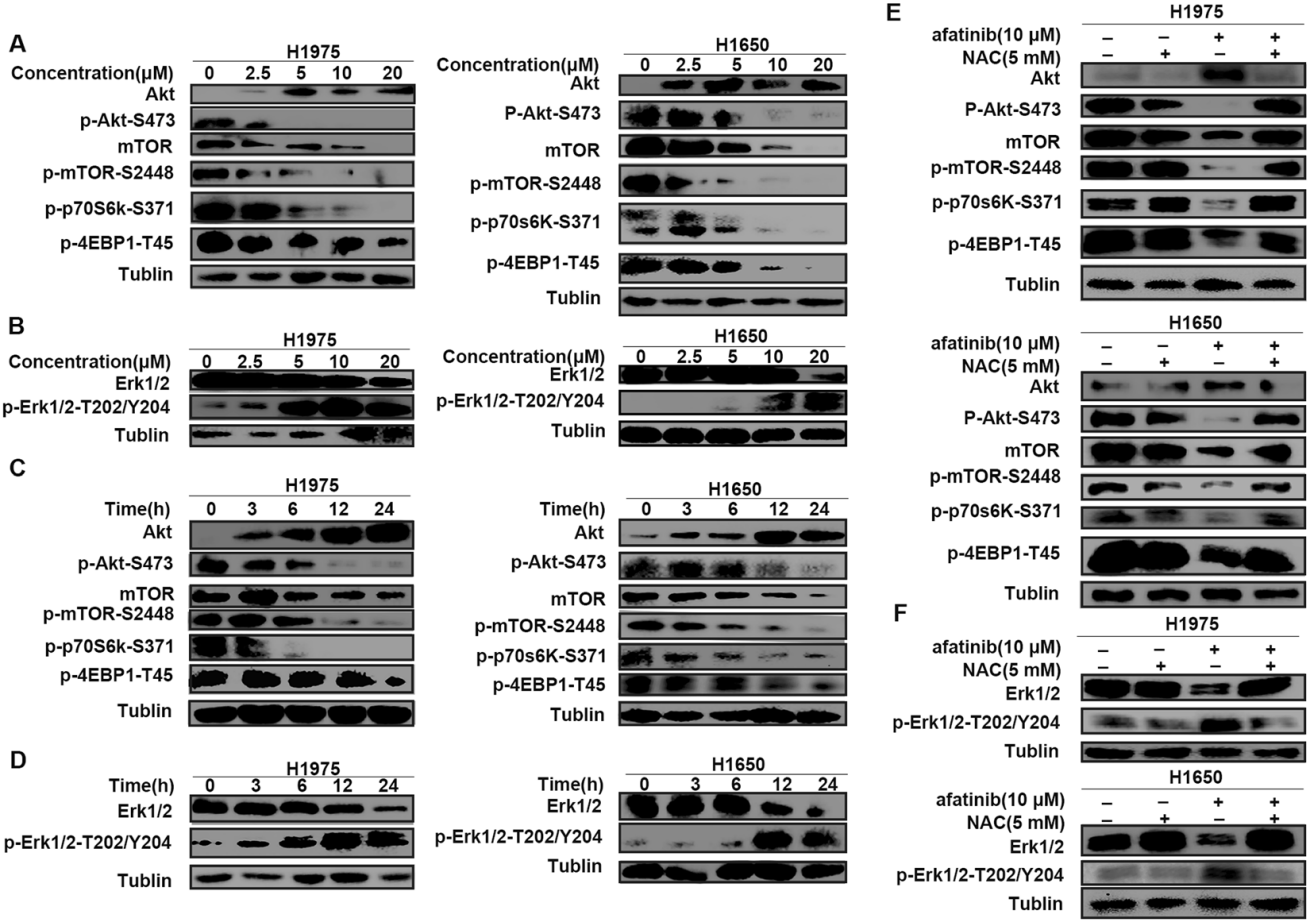
The afatinib-induced autophagy is related to the Akt/mTOR and Erk signaling pathways. (**A** and **B**) H1975 and H1650 cells were stimulated by various concentrations of afatinib for 24 h. (**A**) The levels of Akt, p-Akt, mTOR, p-mTOR, p-P70S6K and p-4EBP1 were analyzed by western blot. (**B**) The levels of Erk 1/2 and p-Erk1/2 were analyzed by western blot. (**C** and **D**) H1975 and H1650 cells were treated with 10 μM afatinib for 0, 3, 6, 12 and 24 h. (**C**) The levels of Akt, p-Akt, mTOR, p-mTOR, p-P70S6K and p-4EBP1 were analyzed by western blot. (**D**) The levels of Erk 1/2 and p-Erk1/2 were detected by western blot. (**E** and **F**) H1975 and H1650 cells were treated with 10 μM afatinib in combination with or without 5 mM of NAC for 24 h. (**E**) The levels of Akt, p-Akt, mTOR, p-mTOR and p-P70S6K were analyzed by western blot. (**F**) The levels of Erk1/2 and p-Erk1/2 were analyzed by western blot. (**A**–**F**) All blots were cropped with Photoshop CS6.

It has been reported that the Akt/mTOR signaling pathway is one of the major signaling pathways acting as a negative regulator of autophagy^31^. To uncover the underlying molecular mechanism of afatinib-induced autophagy, we next investigated the activation status of the key members in Akt/mTOR pathway using western blot analysis. Figure 6A and C indicated that the phosphorylation of key members including Akt, mTOR and its two downstream substrates, p70S6K and eukaryotic initiation factor 4EBP-1 decreased dose- and time-dependently in H1975 and H1650 cells after treatment with afatinib. mTOR can be phosphorylated by its upstream regulator phosphorylated (p)-AKT-serine(S)473 to form p-mTOR-S2448, which negatively regulates autophagy through the phosphorylation of its substrates p70S6K and 4EBP-1^32^. The reduced phosphorylation levels of Akt, mTOR and its substrates indicated that afatinib triggered autophagy via Akt/mTOR signaling pathway.

Extracellular signal-regulated kinase (Erk1/2) is another important pathway involved in autophagy which can regulate the expression of autophagy and lysosomal related genes^33, 34^. Then we investigated the level of phosphorylated Erk1/2 using western blot analysis. A time- and dose-dependent up-regulation of Erk1/2 phosphorylation was observed in H1975 and H1650 cells treated with various concentrations of afatinib for 24 h or 10 μM of afatinib for 3 h, 6 h, 12 h and 24 h (Fig. 6B and D).

These experiment results suggest that the autophagy in afatinib-treated lung adenocarcinoma cells is related to Akt/mTOR and Erk signaling pathway.

### The afatinib-triggered autophagy and growth inhibition are related to intracellular ROS

**Figure 7:**
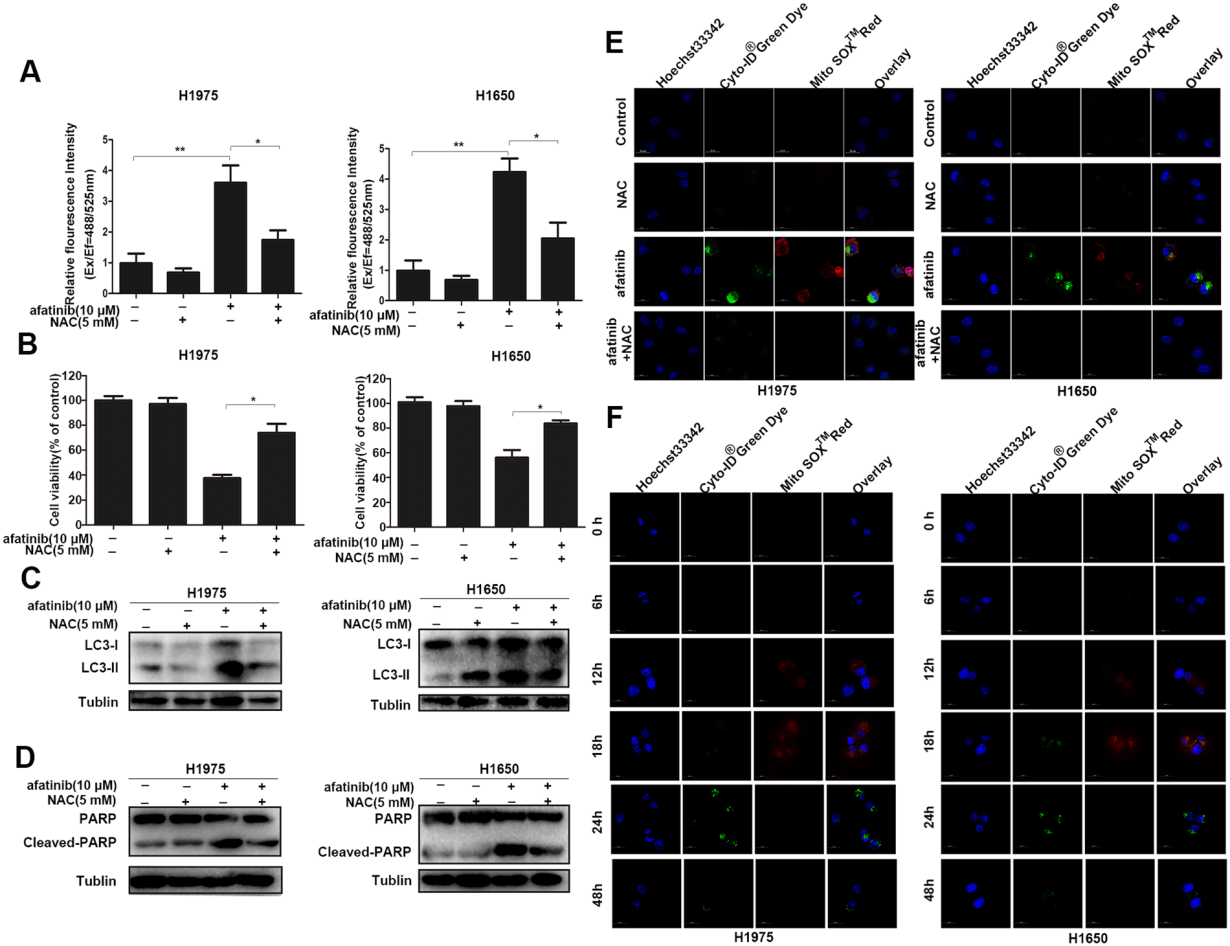
The autophagy and cytotoxicity induced by afatinib are related to intracellular ROS. (**A**–**E**) H1975 and H1650 cells were treated by 10 μM afatinib in the presence or absence of 5 mM NAC for 48 h. (**A**) ROS assay kit was used to analyze intracellular ROS by measuring the DCF fluorescence intensity (*P < 0.05, **P < 0.01). (**B**) Cell viability was analyzed with MTT assay (*P < 0.05). (**C**) The levels of LC3-I/II were measured by western blot analysis. These blots were cropped with Photoshop CS6. (**D**) Western blot analysis was used to detect the level of PARP and Cleaved-PARP. These blots were cropped with Photoshop CS6. (**E**) H1975 and H1650 cells were co-stained with Cyto-ID ^®^Green Dye and Mito Sox^TM^ Red Dye at 37 °C for 15 min, and detected by confocal microscopy. (**F**) After treated with 10 μM afatinib for 0, 6, 12, 18, 24 and 48 h, H1975 and H1650 cells were stained with Cyto-ID ^®^Green Dye and Mito Sox^TM^ Red Dye at 37 °C for 15 min, then analyzed by confocal microscopy.

Many studies prove that reactive oxygen species (ROS) accumulation induced by various stresses serves as a critical intracellular signal transducer sustaining autophagy^35^. To investigate whether ROS was involved in afatinib-induced autophagy, we first detected intracellular ROS accumulation in H1975 and H1650 cells after exposure to 10 μM of afatinib with or without antioxidant N-acetyl-cysteine (NAC). As shown in Fig. 7A, afatinib-treated cells displayed significant ROS accumulation as compared with non-treated cells, and NAC could down-regulate oxidative stress. In addition, MTT assay showed that scavenging ROS by NAC could rescue H1975 and H1650 cells from afatinib-induced growth inhibition (Fig. 7B). Second, western blot assay showed that NAC could decrease afatinib-induced LC3-II and cleaved-PARP accumulations significantly in both cells, suggesting that removing ROS resulted in autophagy inhibition as well as apoptosis down-regulation (Fig. 7C and D).

Furthermore, ROS detection kit Mito SOX^TM^ and autophagy detection kit Cyto-ID^®^ were used to investigate the generation process of intracellular ROS and autophagy. Figure 7E showed that afatinib-treated cells exhibited obvious ROS-specific red fluorescence and autophagosome-specific green fluorescence compared with non-treated cells, whereas cells co-treated with NAC displayed almost no red and green fluorescence. Moreover, as shown in Fig. 7F, after exposure to 10 μM of afatinib for 18 h both cells displayed enhanced red fluorescence indicating ROS generation, then both cells had clear green fluorescence representing autophagy activation in 24 h of afatinib incubation. Additionally, Fig. 6E and F showed that both Akt/mTOR and Erk singal pathways were reversed after scavenging afatinib-induced ROS by NAC.

The results reveal that ROS acts as an intracellular transducer regulating both autophagy and apoptosis in afatinib-treated H1975 and H1650 cells.

### Autophagy suppression improves the anti-tumor effect of afatinib in lung adenocarcinoma xenograft model

**Figure 8:**
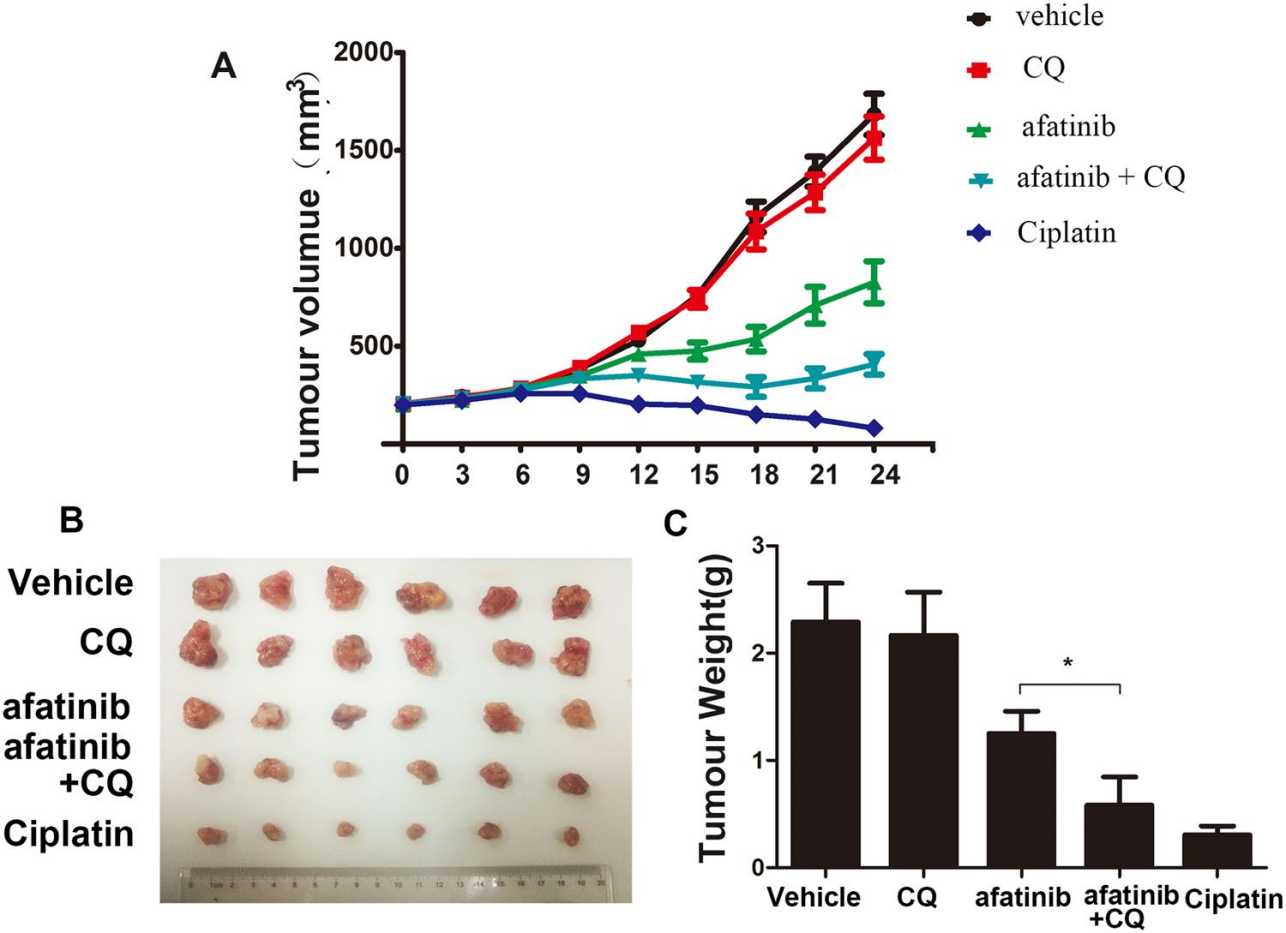
Suppression of autophagy improves the antitumor effect in lung adenocarcinoma xenograft model. (**A**) Athymic nude mice bearing isogenic H1975 xenograft tumors were treated with vehicle, afatinib, CQ, afatinib in combination with CQ and the cisplatin. Tumor growth was measured as described in Materials and Methods. The growth curves represent the average values of 6 mice in each group. Error bars indicate standard deviation. (**B**) The picture of different tumor groups. (**C**) The tumor weight of different groups. All data were presented as mean ± SD (*P < 0.05).

To examine whether blocking autophagy potentiated the afatinib’s anti-lung adenocarcinoma effect *in vivo*, H1975 cells were inoculated into Nu/Nu mice to establish a subcutaneous xenotransplanted tumor model. When the average tumor volume reached 200 mm^3^, the different groups were treated with vehicle (methycellulose/Tween 80), afatinib, CQ, afatinib combined with CQ, and cisplatin (positive control), then their effects on tumor growth were assessed. As expect, afatinib exhibited certain anti-tumor effect *in vivo*. However, as shown in Fig. 8A and B, combination treatment with afatinib and CQ have more remarkable anti-tumor effect than treatment with afatinib alone, and the average values of tumor volume decreased by 427 mm^3^. Moreover, the combination treatment also significantly reduced tumor weight (Fig. 8C).

In brief, these data prove that the suppression of autophagy can improve the anti-tumor effect of afatinib *in vivo*.

## Discussion

NSCLC, especially lung adenocarcinoma, is the commonest fatal malignancy worldwide. The advent of first-generation reversible EGFR-TKIs improved the approaches for NSCLC diagnosis and treatment^36^. Patients with sensitizing *EGFR* mutations show a better prognosis and sensitivity to EGFR-TKIs than conventional chemotherapy. However, the high frequency of *EGFR* mutations in NSCLC confers different efficacy to reversible EGFR-TKIs^12^. Although in sensitizing *EGFR* mutations such as exon 19 deletion, reversible EGFR-TKIs result in longer progression-free survival (PFS) than conventional chemotherapy, the overall survival (OS) benefit was not obtained. Moreover, Treatment with the first-generation EGFR-TKIs show minimal responses in patients with acquired TKI resistance mutations such as T790M mutation^11^.

As an irreversible inhibitor of the ErbB family of tyrosine kinases, the second-generation TKIs afatinib is a valuable option for patients with advanced lung adenocarcinoma harboring activating *EGFR* mutations^37^. Although targeted treatment of these patients with afatinib results in improved anti-tumor responses, acquired resistance is also a major clinical issue in lung cancer therapy with this irreversible pan-ErbB inhibitor^14^. Autophagy is reported to be associated with resistance to afatinib, but its exact effect and mechanisms has not been clearly elucidated especially in lung adenocarcinoma with activating *EGFR* mutations^25^.

In the present study, we chose a L858R/T790M mutant H1975 cell and an exon 19 deletion mutant H1650 cell to investigate the autophagy induction and the effect on anti-tumor activity of afatinib. In addition, the *in vivo* tumor responses were evaluated in H1975 lung adenocarcinoma xenograft model. We first observed growth inhibition and apoptosis in afatinib-treated H1975 and H1650 cells. Many studies prove that the TKI-induced apoptosis is caspase 3 dependent^38, 39^, and it has been reported that afatinib could induce H1975, H1650 and RB116 cell apoptosis by activation of caspase 3^40, 41^. Our experiments proved that afatinib dramatically increased casepase 3 activity, and pan-caspase inhibitor z-VAD-fmk could rescue the cells and decrease caspase 3 activity as well as the level of cleaved-PARP. Therefore, we concluded that afatinib-induced apoptosis was partially caspase 3-dependent in H1975 and H1650 cells.

Autophagy has been considered as an adaptive catabolic process responding to various stress conditions including chemotherapy drugs^42^, and serves as one of important resistance mechanisms to TKI treatment in many cancer cells such as lung cancer, chronic myeloid leukaemia (CML), tongue cancer and pancreatic cancer^25–28^. In this study, we focused on whether afatinib induced autophagy and its role in afatinib’s anti-tumor activity in H1975 and H1650 cells. Through TEM, confocal Immunofluorescence and western blot analysis, we confirmed that afatinib did induce autophagy in lung adenocarcinoma cells with activating *EGFR* mutations^23, 43^.

Chemotherapeutics can initiate either cytoprotective autophagy or autophagic cell death in tumor cells depending on different cell types and microenvironment^38, 44^. We found that suppression of autophagy using two kinds of autophagy inhibitors CQ and 3-MA could significantly enhance afatinib-induced cytotoxicity as well as apoptosis in H1975 and H1650 cells. These observations proved the cytoprotective role of autophagy in afatinib-treated H1975 and H1650 cells, suggesting that abolishing autophagy could enhance the anti-lung adenocarcinoma effect of afatinib *in vitro*. We also established a H1975 cell xenograft model to further confirm the protective effect of autophagy on afatinib therapy *in vivo*. The results displayed that combination treatment with afatinib and CQ enhanced afatinib’s therapeutic efficacy, which further proved that blocking autophagy could overcome resistance against afatinib in lung adenocarcinoma with double mutations.

In the study of the underlying mechanisms of afatinib-induced autophagy, we observed up-regulations of intracellular ROS accumulation and Erk signaling, as well as a down-regulations of Akt/mTOR signaling in H1975 and H1650 cells after afatinib treatment. Intracellular ROS triggered by diverse stress conditions has been considered as critical mediator of autophagy and apoptosis^23, 35^. Through fluorescence microscope, we found that afatinib first initiated intracellular ROS accumulation which then induced autophagy. After scavenging ROS by NAC, the levels of autophagy-related protein LC3-II and apoptosis-related protein cleaved-PARP decreased, and cell viability restored. Therefore, afatinib-triggered ROS not only exerted cytotoxic effect on H1975 and H1650 cells, but also initiated autophagic responses to protect tumor cells from apoptosis and growth inhibition, which can explain our *in vitro* and *in vivo* experiment results that combination treatment with autophagy inhibitors enhanced afatinib’s therapeutic efficacy.

Besides, antioxidant NAC also reversed afatinib-induced changes of Akt/mTOR and Erk signaling pathway. Previous studies reported that the Akt/mTOR signaling pathway negatively regulates autophagy through phosphorylation of Akt, downstream effector mTOR and its substrates p70S6K and 4E-BP1^45^, and Erk can be activated through phosphorylation of threonine and tyrosine residues, then induces the formation of cytoplasmic macrovacuoles, a symbol of autophagy^46^. In the present study, western blot analyses demonstrated that both Akt/mTOR and Erk signaling pathways were involved in afatinib-induced autophagy in H1975 and H1650 cells, and what’s more, afatinib-triggered ROS accumulation initiated autophagic responses through influencing Akt/mTOR and Erk signaling pathways.

In conclusion, our research proves that afatinib can induce cytotoxicity as well as autophagy in H1975 and H1650 cells. More importantly, inhibition of autophagy enhances the cytotoxicity of afatinib *in vitro*, suggesting the cytoprotective role of afatinib-induced autophagy. Further study suggests that Akt/mTOR and Erk signaling pathways are involved in autophagy triggered by afatinib, and intracellular ROS acts as a critical mediator of autophagy and cytotoxicity. Additionally, we demonstrate that autophagy suppression can potentiate the therapeutic efficacy of afatinib *in vivo*. Our research highlights that combination of afatinib and autophagy inhibitor may be a promising approach to increase the sensitivity of lung adenocarcinoma cells harboring activating *EGFR* mutations to afatinib treatment.

## Methods

### Cell lines and culture

The lung adenocarcinoma cells H1975 and H1650 were grown in RPMI-1640 (CORNING) medium containing 10% fetal bovine serum (Capricon), 100 IU/ml of penicillin and 100 g/ml of streptomycin (Beyotime Institute of Biotechnology). All cells were cultured in 5% CO_2_ at 37 °C.

### Cell viability assay

MTT assay was used to detect the cell viability. About 1 × 10^4^ cells per well were planted in 96-well plates and then exposed to CQ or 3-MA (Sigma-Aldrich) for 2 hours, then added the indicated concentrations of afatinib (Shanghai Hanxiang Biotech) into different wells. After certain time, cells were incubated with MTT solution (0.5 mg/ml) (Beyotime Institute of Biotechnology) for 4 h at 37 °C. Then 150 μl DMSO was added to each well to dissolve formazan for measurement. The optical density was recorded at the wavelength of 570 nm by microplate reader.

### Confocal immunofluorescence

About 1.8 × 10^5^/ml of H1975 or H1650 cells were planted in 6-well plates. Some wells were added with 10 μM afatinib for 24 h, or treated with 50 nM rapamycin (Sigma-Aldrich) for 6 h as positive controls, and the left wells were used as negative control. After that, cells were washed twice by culture medium and then added with Cyto-ID^®^ green dye and nuclear dye Hoechst 33342 (Enzo Life Sciences), or ROS detection kit Mito Sox^TM^ red dye (Invitrogen) at 37 °C for 25 min. Cells were immediately detected with fluorescence microscope after incubation. These procedures were done in the dark room.

### Western blot analysis

Cells were washed with cold phosphate-buffered saline (PBS) after added to different concentrations of afatinib for different time. Then, cells were lysed by RIPA buffer (Beyotime Institute of Biotechnology) on ice for 30 min. The lysates were centrifuged at 12,000 rpm at 4 °C for 10 min, and the supernatant was collected. Then the lystates protein was quantized by BCA protein quantitation kit (Beyotime Institute of Biotechnology). The equal amount of protein per lane was separated by SDS-PAGE, then the protein was transferred to PVDF membranes. After blocked by 3% bovine saline albumin, the membranes were washed with 1 × TBST (150 mM NaCl, 10 mM TRIS-Cl, pH 8.0), containing 0.05% (v/v) Tween-20 (Dalian Meilun Biotech) for three times. After incubated overnight with specialized primary antibodies (Cell Signaling), membranes were washed for three times with 1 × TBST and suspended in the secondary antibodies buffer (Beyotime Institute of Biotechnology) for 2 h at room temperature. At last detection was performed with enhanced chemiluminescence reagents (Beyotime Institute of Biotechnology).

### Transmission electron microscopy analysis

H1650 and H1975 cells were incubated with or without afatinib (10 μM) for 24 h. The harvested cells were fixed in ice-cold glutaraldehyde, then sliced and analyzed by a JEM 1410 transmission electron microscope (JEOL, Inc., USA). Micrographs were taken at 5 × 10^3^ or 2 × 10^4^ magnification.

### Detection of intracellular ROS

Intracellular ROS production was analyzed in H1650 and H1975 cells with ROS assay kit (Beyotime Institute of Biotechnology). ROS can oxidize dichlorofluorescein diacetate (DCFH-DA), a cell infiltrative and non-fluorescent dye, to fluorescent carboxy dichlorofluorescein. H1650 and H1975 cells were co-incubated with afatinib (10 μM) and 10 μM DCFH-DA at 37 °C for indicated time, then washed twice by PBS. The Tecan Infinite^®^ 200 PRO microplate reader was used to quantify DCF fluorescence intensity at excitation wavelength of 488 nm and emission wavelength of 525 nm.

### Caspase 3 activity analysis

After treated with specific concentrations of afatinib in the presence or absence of autophagy inhibitors, H1650 and H1975 cells were lysed and the activity of apoptosis-related protein caspase 3 was measured by Caspase 3 Activity detection kit (Beyotime Institute of Biotechnology).

### Tumor xenograft model

H1975 cells (1 × 10^6^/ml) were inoculated subcutaneously into the right thigh of male 4 to 6 weeks old of Nu/Nu mice (Charles River). When the average tumor volume reached 200 mm^3^, the mice were randomized into 5 groups to receive the following treatments: (a) vehicle (methycellulose/Tween 80, per os) (Dalian Meilun Biotech); (b) CQ (50 mg/kg, intraperitoneal injection); (c) afatinib (20 mg/kg/day, per os); (d) afatinib (20 mg/kg/day, per os) + CQ (50 mg/kg, intraperitoneal injection) (e) cisplatin (10 mg/kg, every other day, intraperitoneal injection) (Dalian MeilunBiotech). Tumors were measured every 3 days, and tumor volumes were determined from caliper measurements of tumor length (*L*) and width (*W*) according to the formula *V *=  (*L* × *W* ^2^)/2. All animal procedures were performed in accordance with institutional guidelines and with approval from Fudan University Committee on Animal Care.

### Statistical analysis

Statistical analysis was performed by GraphPad Prism 5 and the data were presented as mean ± S.D. The two-tailed Student’s t test was used to compare multiple sets of data. *P* < 0.05 were considered to be statistically remarkable.
